# Implementation of contact precautions for multidrug-resistant organisms in the post–COVID-19 pandemic era: An updated national Emerging Infections Network (EIN) survey

**DOI:** 10.1017/ice.2024.11

**Published:** 2024-06

**Authors:** Jessica R. Howard-Anderson, Lindsey B. Gottlieb, Susan E. Beekmann, Philip M. Polgreen, Jesse T. Jacob, Daniel Z. Uslan

**Affiliations:** 1 Division of Infectious Diseases, Department of Medicine, Emory University School of Medicine, Atlanta, Georgia; 2 Division of Infectious Diseases, Department of Internal Medicine, University of Iowa Carver College of Medicine, Iowa City, Iowa; 3 Division of Infectious Diseases, Department of Medicine, David Geffen School of Medicine, University of California– Los Angeles, Los Angeles, California

## Abstract

**Objective:**

To understand how healthcare facilities employ contact precautions for patients with multidrug-resistant organisms (MDROs) in the post–coronavirus disease 2019 (COVID-19) era and explore changes since 2014.

**Design:**

Cross-sectional survey.

**Participants:**

Emerging Infections Network (EIN) physicians involved in infection prevention or hospital epidemiology.

**Methods:**

In September 2022, we sent via email an 8-question survey on contact precautions and adjunctive measures to reduce MDRO transmission in inpatient facilities. We also asked about changes since the COVID-19 pandemic. We used descriptive statistics to summarize data and compared results to a similar survey administered in 2014.

**Results:**

Of 708 EIN members, 283 (40%) responded to the survey and 201 reported working in infection prevention. A majority of facilities (66% and 69%) routinely use contact precautions for methicillin-resistant *Staphylococcus aureus* (MRSA) and vancomycin-resistant enterococci (VRE) respectively, compared to 93% and 92% in 2014. Nearly all (>90%) use contact precautions for *Candida auris*, carbapenem-resistant Enterobacterales (CRE), and carbapenem-resistant *Acinetobacter baumannii*. More variability was reported for carbapenem-resistant *Pseudomonas aeruginosa* and extended-spectrum β-lactamase–producing gram-negative organisms. Compared to 2014, fewer hospitals perform active surveillance for MRSA and VRE. Overall, 90% of facilities used chlorhexidine gluconate bathing in all or select inpatients, and 53% used ultraviolet light or hydrogen peroxide vapor disinfection at discharge. Many respondents (44%) reported changes to contact precautions since COVID-19 that remain in place.

**Conclusions:**

Heterogeneity exists in the use of transmission-based precautions and adjunctive infection prevention measures aimed at reducing MDRO transmission. This variation reflects a need for updated and specific guidance, as well as further research on the use of contact precautions in healthcare facilities.

Contact precautions, a category of transmission-based precautions, require healthcare personnel to don a gown and gloves prior to entering a patient’s room.^1^ Based on the 2007 Healthcare Infection Control Practice Advisory Committee (HICPAC) Guidelines, the Centers for Disease Control and Prevention (CDC) recommends routine use of contact precautions when caring for patients with multidrug-resistant organisms (MDROs).^1,2^ In this document, MDROs are defined as microorganisms resistant to 1 or more classes of antimicrobial agents, but they are often resistant to most available antimicrobial agents. The widely referenced CDC “Appendix A: Type and Duration of Precautions Recommended for Selected Infections and Conditions” comments that MDROs should be “of clinical and epidemiologic significance” and includes methicillin-resistant *Staphylococcus aureus* (MRSA), vancomycin-resistant enterococci (VRE), extended-spectrum β-lactamase (ESBL)–producing organisms, and resistant *Streptococcus pneumoniae* as examples.^3^

Which MDROs should require contact precautions is unclear and frequently debated.^4–7^ Much of the data referenced in the 2007 HICPAC guidelines evaluated the impact of contact precautions on MDRO transmission as part of larger infection-prevention bundles, making it difficult to assess the relative contribution of contact precautions.^1,2^ Prior to the coronavirus disease 2019 (COVID-19) pandemic, some healthcare facilities stopped routinely recommending contact precautions for MRSA and VRE and did not see an increase in healthcare associated infections (HAIs) due to these organisms.^8–10^ However, many of these studies were conducted at a single center and were limited by quasi-experimental designs. Others have argued that contact precaution policies specifically for MRSA have played a large role in the significant decline in prepandemic MRSA rates both in the Veterans’ Affairs (VA) system and nationally.^11–13^ In the Benefits of Universal Glove and Gown (BUGG) cluster-randomized controlled trial, universal gown-and-glove use was associated with decreased MRSA acquisition and no increase in adverse events.^14^ Even less is known about the impact and utility of contact precautions on the transmission of multidrug-resistant gram-negative organisms. A cluster-randomized crossover trial in 4 European acute-care hospitals, did not demonstrate a reduction in ESBL-producing Enterobacterales carriage with the addition of contact precautions. At least 1 healthcare facility has discontinued contact precautions for ESBL-producing organisms and has shown similar findings.^15,16^

During the coronavirus disease 2019 (COVID-19) pandemic, shortages in personal protective equipment (PPE), and the limited availability of single-occupant patient rooms in some facilities forced many hospitals to reconsider the use of contact precautions. We hypothesized that COVID-19 may have been the initial impetus to discontinue contact precautions for MRSA or other MDROs but that many facilities did not return to prepandemic contact precautions polices, even after the supply shortage ended. Using the Emerging Infections Network (EIN), we surveyed clinicians involved in infection prevention or hospital epidemiology about which MDROs require contact precautions in their facility and what adjunctive measures are employed to minimize MDRO transmission. We compared our results to a similar survey that was administered through the EIN in 2014.^17^

## Methods

The EIN is a CDC-funded cooperative program that serves as a “sentinel network” of infectious disease clinicians to help detect, identify, and gather information on emerging infectious diseases.^18^ All authors reviewed the original 2014 EIN survey on contact precautions,^17^ and through iterative feedback, agreed on the revised 2022 version. The updated 8-question survey asked about the respondent’s primary inpatient healthcare facility’s (self-defined) recommendations on transmission-based precautions and adjunctive measures employed to reduce MDRO transmission. Compared to the 2014 survey, we added questions related to multidrug-resistant gram-negative organisms, *Candida auris*, and the impact of the COVID-19 pandemic. The questions included both discrete answer choices as well as free-text responses. The full survey is included in Supplementary Materials (online).

On September 8, 2022, we distributed the survey via email to the EIN physician members who had reported infection prevention or hospital epidemiology responsibilities. According to EIN protocol, we excluded physician members who had never answered any EIN surveys from the denominator when reporting results. We sent 2 reminder emails over the following month. We used descriptive statistics to summarize our results and, where applicable, we compared our results to the 2014 survey.

## Results

Of the 708 EIN members with reported infection prevention or hospital epidemiology responsibilities, 283 (40%) responded to the survey. Most respondents were adult infectious diseases physicians (80%) with at least 15 years of experience (63%). Nearly all the respondents worked in the United States (99%), with relatively equal geographic distribution. Similar proportions of respondents reported working in community (25%), university (36%) and nonuniversity teaching facilities (29%) (Table 1). Of the initial 283 respondents, 201 (71%) reported being involved in infection prevention, and they completed at least 1 of the remaining survey questions. Another 82 (29%) were not involved in infection prevention and were excluded from the remaining survey.

**Table 1.** Characteristics of All Survey Respondents

Characteristic	No. (%) (N=283)
**Field of practice**	
Adult infectious diseases	226 (80)
Pediatric infectious diseases	57 (20)
**Region**	
Midwest US	75 (27)
South US	72 (25)
West US	67 (24)
Northeast US	66 (23)
Canada and Puerto Rico	3 (1)
**Experience since ID fellowship**	
<5 y	43 (15)
5–14 y	66 (23)
15–24 y	59 (21)
≥25 y	115 (41)
**Primary facility type**	
University hospital	102 (36)
Non-university teaching hospital	83 (29)
Community hospital	67 (24)
VA or DOD hospital	15 (5)
City/county hospital	15 (5)
Outpatient only^a^	1 (0.4)

Note. DOD, Department of Defense; VA, Veterans’ Affairs.

a This respondent answered the survey questions based on the policies of the inpatient facility that was connected to ambulatory clinic.

**Figure 1.**
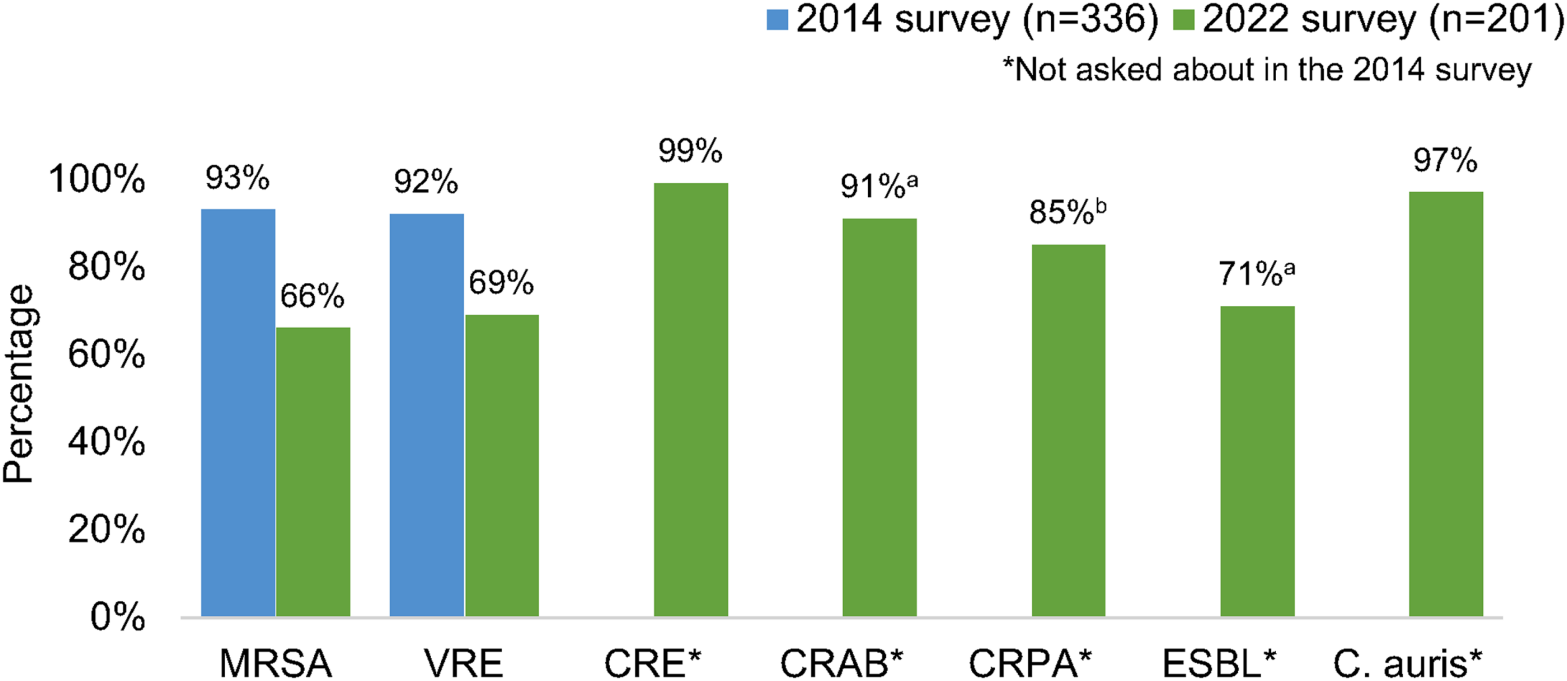
Percentage of respondents whose primary facility uses contact precautions for selected multidrug-resistant organisms. (a) Answered by 196 respondents. (b) Answered by 192 respondents. Note. MRSA, methicillin-resistant *Staphylococcus aureus*; VRE, vancomycin-resistant enterococci; CRE, carbapenem-resistant Enterobacterales; CRAB, Carbapenem-resistant *Acinetobacter baumannii*; CRPA, Carbapenem-resistant *Pseudomonas aeruginosa;* ESBL, extended-spectrum β-lactamase–producing organisms.

Most respondents reported that their facility routinely used contact precautions for MRSA (66%) and VRE (69%), which decreased from 93% and 92%, respectively, in 2014.^17^ Nearly all (>90%) reported requiring contact precautions for *C. auris*, carbapenem-resistant Enterobacterales (CRE), and carbapenem-resistant *Acinetobacter baumannii*. More variability was reported in the use of contact precautions for carbapenem-resistant *Pseudomonas aeruginosa* and ESBL-producing organisms (Fig. 1). Recommendations for contact precautions appeared similar between community and academic healthcare settings. Nearly 100% of respondents working in VA hospitals reported using contact precautions for MRSA, VRE, CRE and *C. auris* (Supplementary Table 1 online).

**Figure 2.**
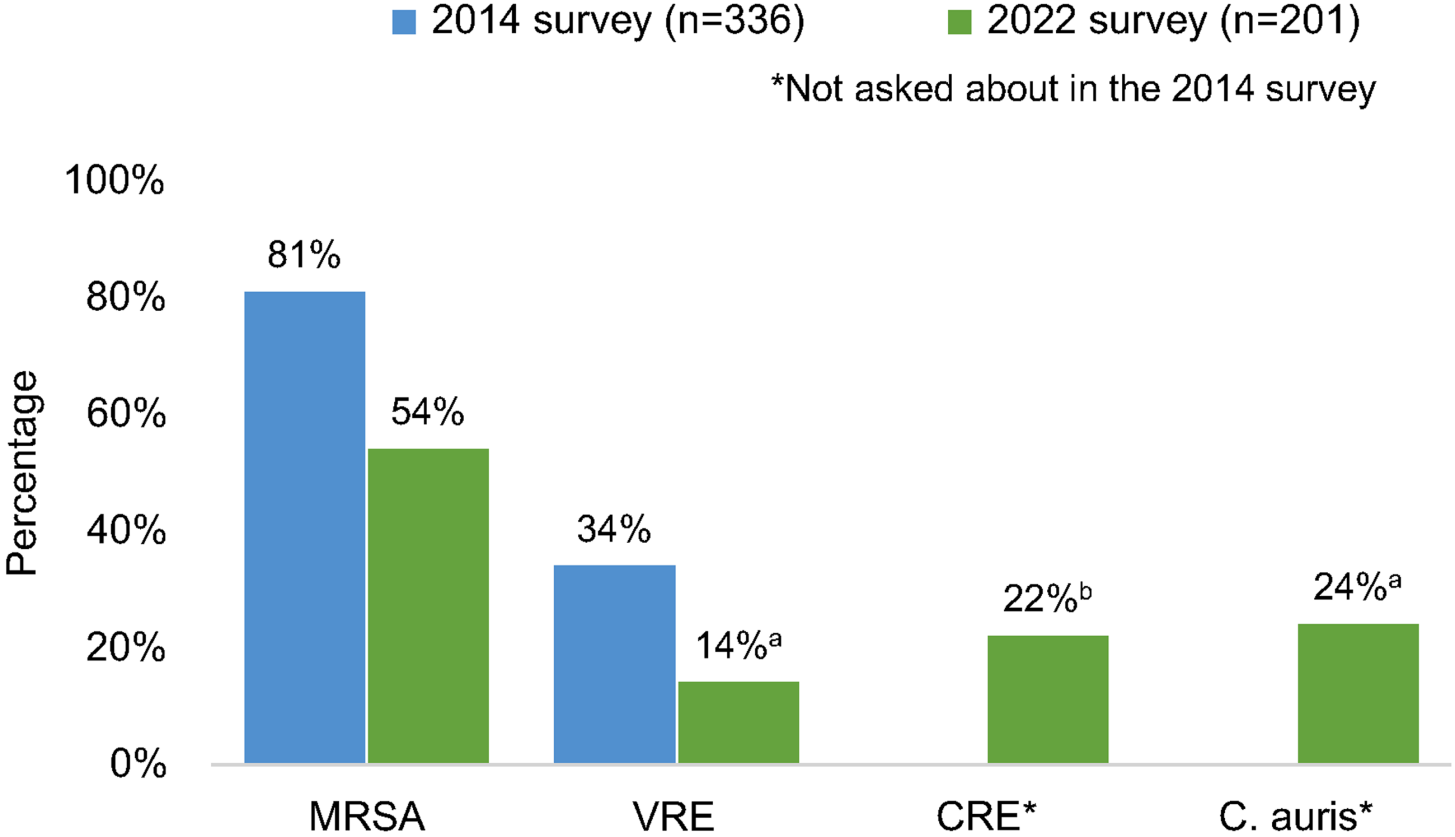
Percentage of respondents whose primary facility performs active surveillance for selected multidrug-resistant organism. (a) Answered by 197 respondents. (b) Answered by 196 respondents. Note. MRSA, methicillin-resistant *Staphylococcus aureus;* VRE, vancomycin-resistant enterococci; CRE, carbapenem-resistant Enterobacterales.

Active surveillance for MRSA (54%) was still performed more frequently than for other MDROs (including VRE, CRE or *C. auris*) but was lower than reported in 2014 (81%). Active surveillance for VRE also decreased from 2014 to 2022 (Fig. 2). The duration of contact precautions employed varied by organism (Table 2). Compared to 2014, facilities in this survey were less likely to use contact precautions indefinitely for MRSA (18% vs 6%) and VRE (31% vs 11%).^17^ For CRE and *C. auris,* >75% of respondents reported that their facility either used contact precautions indefinitely or only removed contact precautions if the patient was “cleared or decolonized” (Table 2).

**Table 2.** Duration of Contact Precautions Once a Patient Is Identified to Have a Multidrug-Resistant Organism

Organism	No. (%) of Respondents			
	Indefinitely Once Positive	Until Cleared or Decolonized	For 1 Year After Last Positive Culture	For Specific Inpatient Encounter Only
MRSA^a^	11 (6)	73 (38)	25 (13)	34 (18)
VRE^b^	21 (11)	53 (29)	3 (16)	28 (15)
CRE^c^	97 (51)	50 (26)	30 (16)	21 (11)
*C. auris* ^b^	117 (62)	32 (17)	16 (8)	16 (8)

Note. MRSA, methicillin-resistant *Staphylococcus aureus*; VRE, vancomycin-resistant enterococci; CRE, carbapenem-resistant Enterobacterales.

a Answered by 190 participants.

b Answered by 183 participants.

c Answered by 191 participants.

Most facilities (90%) performed chlorhexidine gluconate (CHG) bathing either in all inpatients or a subset of inpatients (Table 3). Compared to the 2014 survey, more respondents in the 2022 survey reported using CHG bathing on all inpatients (7% in 2014 vs 19% in 2022).^17^ Similarly, for environmental cleaning, more respondents reported using ultraviolet light or hydrogen peroxide vapor disinfection at time of any patient discharge (23% in 2014 vs 53% in 2022). Adenosine triphosphate bioluminescence assays were the most common technique for monitoring inpatient environmental cleaning (50%) (Table 4).

**Table 3.** Number of Respondents Whose Facility Performs CHG Bathing on the Following Inpatient Populations

Population	No. (%) of Respondents	
	2014 Survey (n = 354)	2022 Survey (n = 199)
None in inpatients	29 (8)	19 (10)
All inpatients unless contraindicated	25 (7)	38 (19)
Subset of inpatients^a,b^	300 (85)	142 (71)
Intensive care unit		108 (76)^c^
Surgical/pre-operative^d^		101 (71)^c^
Patients with central lines or other implants		81 (57)^c^
Pediatrics		20 (14)^c^
Oncology		18 (13)^c^
Other		10 (7)^c^

a Respondents could select all subsets that applied.

b The 2014 survey included different options than the 2022 survey, so data on the 2014 subsets were not include here.

c Denominator for the percentage is 142.

d Respondents could select only a subset of surgical patients to whom this applied.

**Table 4.** Number of Respondents whose Facility Routinely Uses the Following Practices for Monitoring Environmental Cleaning

Practice Used	No. (%) of Respondents	
	2014 Survey (n = 335)	2022 Survey (n = 201)
ATP bioluminescence	145 (43)	100 (50)
Visual inspection	167 (50)	89 (44)
Blacklight inspection	74 (22)	56 (28)
Unsure	72 (22)	43 (21)
Do not monitor	34 (10)	9 (4)

Note. ATP, adenosine triphosphate. Respondents were instructed to select all practices that applied.

Lastly, many (44%) reported institutional changes to contact precautions policies after the start of the COVID-19 pandemic that remained in place at the time of the survey. Furthermore, 60% did not anticipate their contact precautions practices changing in the next year.

## Discussion

In this nationwide survey of >200 experienced physicians with expertise in infection prevention, contemporary use of contact precautions was heterogeneous and varied by the MDRO. Although routine contact precautions for MRSA or VRE were used in hospitals for >66% of the survey respondents, this rate notably decreased from 2014 when >90% reported using contact precautions for these pathogens. We also observed a similar decline in active surveillance for these gram-positive organisms, and active surveillance for any pathogen was rare. Contact precautions were nearly universally recommended for CRE and *C. auris*; however, variability existed in the recommendations for other multidrug-resistant gram-negative pathogens.

Recently, the Society for Healthcare Epidemiology of America (SHEA), in partnership with the Infectious Diseases Society of America (IDSA) and the Association for Professionals in Infection Control and Epidemiology (APIC), published updated guidance on strategies to prevent MRSA transmission in acute-care hospitals, recommending use of contact precautions for all patients infected or colonized with MRSA.^13^ Based on our survey, a significant proportion of facilities will now be nonadherent to this guidance, but it remains to be seen whether this SHEA update will prompt more facilities to return to prepandemic policies. The SHEA guidance acknowledges that not all hospitals are routinely using contact precautions for MRSA and includes guidance about when deviation from this approach could be considered, in the setting of performing a risk assessment, monitoring MRSA rates, and ensuring other horizontal infection prevention practices are in place to prevent MDRO transmission. Our survey did not exhaustively assess every horizontal or adjunctive infection prevention measure for MRSA, and notably, we did not ask about hand-hygiene-monitoring programs. The survey results indicated that many facilities are employing CHG bathing for a large subset of hospitalized patients. There may be room for improvement in using environmental cleaning and auditing to minimize MRSA transmission, as >20% of our respondents, who have expertise in infection prevention, were unsure if their facility routinely monitored environmental cleaning.

Although facilities appear to use contact precautions more consistently for multidrug-resistant gram-negative organisms compared to gram-positive organisms, even fewer data are available on the benefit of contact precautions for gram-negative organisms.^19,20^ Policy decisions for gram-negative organisms are particularly challenging as they often consider both molecular and phenotypic definitions of resistance. The CDC toolkit on CRE recommends contact precautions for CRE, although it acknowledges that some institutions will only do this for carbapenemase-producing isolates.^21^ Whether or not contact precautions should be used for other carbapenem-resistant organisms like *P. aeruginosa*, which is unlikely to be carbapenemase-producing in the United States, is unknown.^22^ The lack of unified policy on this and heterogeneity in practice patterns throughout the United States may confuse patients, staff, and clinicians without specific infection-control expertise when they move between multiple facilities. One potential solution may be to transform the SHEA pathogen-specific MRSA guidance into more universal MDRO guidance given the number of horizontal interventions in the MRSA guidance that may be broadly applicable to other MDROs. This guidance document could recommend shared infection prevention strategies that would be effective across different MDROs, while also highlighting any unique considerations of each MDRO included.

The COVID-19 pandemic fundamentally changed the landscape of infection prevention and hospital epidemiology. Although many initial changes to contact precautions policies in early 2020 were due to an urgent need to conserve gowns and gloves, in late 2022, >40% of respondents said changes to contact precautions remained in place. During the pandemic, HAIs increased, including hospital-onset MRSA infections.^23–25^ Although this rise in HAIs is multifactorial, it brings into question whether the tail end of a pandemic is the ideal time to relax contact precautions policies. Additionally, our survey did not ask about COVID-19, but a few respondents added in free-text comments calling for the removal of contact precautions for COVID-19, which is still recommended by the CDC even though evidence of transmission of SARS-CoV-2 via contaminated surfaces or fomites is minimal.^26,27^ It's possible that the continued recommendation for use of contact precautions for COVID-19 without perceived benefit by some healthcare personnel has contributed to negative attitudes toward the use of contact precautions for MDROs.

This study was strengthened by surveying experienced clinicians with expertise in infection prevention throughout the country. Because the survey respondents work in diverse settings including academic and community hospitals, our results are likely representative of most facilities across the country. However, we did not capture facilities that do not have physicians engaged in hospital epidemiology or infection prevention.

The study also had several limitations. First, respondents who work at facilities where contact precautions policies had recently changed may have been more likely to respond to the survey. However, the use of contact precautions is frequently debated among infection-prevention professionals, so major differences between respondents and nonrespondents are unlikely. Second, all our data were self-reported and, thus, were subject to bias. We did not ask respondents to include the name of their primary facility, and duplicate data may have been submitted from the same facility. Third, the survey was cross-sectional, and we could not directly compare individual or facility responses between 2014 and 2022. Fourth, we know some states and health systems require contact precautions for MRSA, so practice may not necessarily reflect attitudes or beliefs of the institution’s infection preventionists or hospital epidemiologist. Lastly, we were unable to determine whether the decline in contact precautions was specifically due to the COVID-19 pandemic; use of contact precautions may have already been declining after 2014 but prior to the pandemic.

In conclusion, we found large variation in institutional infection prevention policies aimed at preventing MDRO transmission in healthcare facilities. We believe that updated and more specific public health guidance defining which organisms require contact precautions and for what duration could help reduce this heterogeneity. Guidance documents may also benefit from including alternative recommendations or considerations for when facilities are not able to implement all recommended infection prevention measures. To inform and iteratively refine such guidance, well-designed trials are needed to investigate the benefits and any potential harms of contact precautions for prevention of MDRO transmission. As we look to the future and emerge from the COVID-19 pandemic, many healthcare organizations are struggling with burnout, workplace violence, and staffing shortages.^28^ Our findings suggest a need to critically re-evaluate how contact precautions are used, which measures are the most effective to prevent the transmission of infectious organisms, and how we can ensure the safety of patients and healthcare personnel.

## Supporting information

Howard-Anderson et al. supplementary material 1 Howard-Anderson et al. supplementary material

Howard-Anderson et al. supplementary material 2 Howard-Anderson et al. supplementary material

## Supplementary material

To view supplementary material for this article, please visit https://doi.org/10.1017/ice.2024.11

## References

[1] Siegel JD , Rhinehart E , Jackson M , Chiarello L. 2007 Guideline for isolation precautions: preventing transmission of infectious agents in healthcare settings. Am J Infect Control 2007;35:S65–S164. 18068815 10.1016/j.ajic.2007.10.007 PMC7119119

[2] Siegel JD , Rhinehart E , Cic R , Jackson M. Management of multidrug-resistant organisms in healthcare settings. Am J Infect Control 2007;35(10 suppl 2):S165–S193. 18068814 10.1016/j.ajic.2007.10.006

[3] Type and duration of precautions recommended for selected infections and conditions (Appendix A). Centers for Disease Control and Prevention website. https://www.cdc.gov/infectioncontrol/guidelines/isolation/appendix/type-duration-precautions.html. Updated 2018. Accessed August 4, 2023.

[4] Young K , Doernberg SB , Snedecor RF , Mallin E. Things we do for no reason: contact precautions for MRSA and VRE. J Hosp Med 2019;14:178–180. 30811326 10.12788/jhm.3126

[5] Morgan DJ , Wenzel RP , Bearman G. Contact precautions for endemic MRSA and VRE: time to retire legal mandates. JAMA 2017;318:329–330. 28654976 10.1001/jama.2017.7419

[6] Goto M , Harris AD , Perencevich EN. Contact precautions and methicillin-resistant *Staphylococcus aureus*—modeling our way to safety. JAMA Network Open 2021;4:e211574. 33720366 10.1001/jamanetworkopen.2021.1574

[7] Bearman GM , Harris AD , Tacconelli E. Contact precautions for the control of endemic pathogens: finding the middle path. Antimicrob Steward Healthc Epidemiol 2023;3:e57. 37008747 10.1017/ash.2023.145 PMC10052432

[8] Haessler S , Martin EM , Scales ME , et al. Stopping the routine use of contact precautions for management of MRSA and VRE at three academic medical centers: an interrupted time series analysis. Am J Infect Control 2020;48:1466–1473. 32634537 10.1016/j.ajic.2020.06.219

[9] Bearman G , Abbas S , Masroor N , et al. Impact of discontinuing contact precautions for methicillin-resistant *Staphylococcus aureus* and vancomycin-resistant *Enterococcus*: an interrupted time series analysis. Infect Control Hosp Epidemiol 2018;39:676–682. 29580304 10.1017/ice.2018.57

[10] Martin EM , Russell D , Rubin Z , et al. Elimination of routine contact precautions for endemic methicillin-resistant *Staphylococcus aureus* and vancomycin-resistant *Enterococcus*: a retrospective quasi-experimental study. Infect Control Hosp Epidemiol 2016;37:1323–1330. 27457254 10.1017/ice.2016.156 PMC6783805

[11] Rubin MA , Samore MH , Harris AD. The importance of contact precautions for endemic methicillin-resistant *Staphylococcus aureus* and vancomycin-resistant enterococci. JAMA 2018;319:863–864. 29435582 10.1001/jama.2017.21122

[12] Jain R , Kralovic SM , Evans ME , et al. Veterans’ Affairs initiative to prevent methicillin-resistant *Staphylococcus aureus* infections. N Engl J Med 2011;364:1419–1430. 21488764 10.1056/NEJMoa1007474

[13] Popovich KJ , Aureden K , Ham DC , et al. SHEA/IDSA/APIC practice recommendation: strategies to prevent methicillin-resistant *Staphylococcus aureus* transmission and infection in acute-care hospitals: 2022 update. Infect Control Hosp Epidemiol 2023;44:1039–1067. 37381690 10.1017/ice.2023.102 PMC10369222

[14] Harris AD , Pineles L , Belton B , et al. Universal glove and gown use and acquisition of antibiotic-resistant bacteria in the ICU: a randomized trial. JAMA 2013;310:1571–1580. 24097234 10.1001/jama.2013.277815 PMC4026208

[15] Maechler F , Schwab F , Hansen S , et al. Contact isolation versus standard precautions to decrease acquisition of extended-spectrum β-lactamase–producing Enterobacterales in noncritical care wards: a cluster-randomised crossover trial. Lancet Infect Dis 2020;20:575–584. 32087113 10.1016/S1473-3099(19)30626-7

[16] Gottlieb LB , Walits E , Patel G , Schaefer S. Taking off the gown: impact of discontinuing contact precautions for extended-spectrum β-lactamase (ESBL)–producing organisms. Antimicrob Steward Healthc Epidemiol 2021;1:e31. 36168477 10.1017/ash.2021.189 PMC9495408

[17] Russell D , Beekmann SE , Polgreen PM , Rubin Z , Uslan DZ. Routine use of contact precautions for methicillin-resistant *Staphylococcus aureus* and vancomycin-resistant *Enterococcus*: which way is the pendulum swinging? Infect Control Hosp Epidemiol 2016;37:36–40. 26486272 10.1017/ice.2015.246

[18] Pillai SK , Beekmann SE , Santibanez S , Polgreen PM. The Infectious Diseases Society of America Emerging Infections Network: bridging the gap between clinical infectious diseases and public health. Clin Infect Dis 2014;58:991–996. 24403542 10.1093/cid/cit932 PMC4634883

[19] Harris AD , Morgan DJ , Pineles L , Magder L , O’Hara LM , Johnson JK. Acquisition of antibiotic-resistant gram-negative bacteria in the Benefits of Universal Glove and Gown (BUGG) cluster randomized trial. Clin Infect Dis 2021;72:431–437. 31970393 10.1093/cid/ciaa071 PMC7850534

[20] Derde LPG , Cooper BS , Goossens H , et al. Interventions to reduce colonisation and transmission of antimicrobial-resistant bacteria in intensive care units: an interrupted time series study and cluster randomised trial. Lancet Infect Dis 2014;14:31–39. 24161233 10.1016/S1473-3099(13)70295-0 PMC3895323

[21] Facility guidance for control of carbapenem-resistant Enterobacteriaceae (CRE). Centers for Disease Control and Prevention website. https://www.cdc.gov/hai/pdfs/Cre/CRE-Guidance-508.pdf. Published 2015. Accessed August 4, 2023.

[22] Reyes J , Komarow L , Chen L , et al. Global epidemiology and clinical outcomes of carbapenem-resistant *Pseudomonas aeruginosa* and associated carbapenemases (POP): a prospective cohort study. Lancet Microbe 2023;4:e159–e170. 36774938 10.1016/S2666-5247(22)00329-9 PMC10016089

[23] Witt LS , Howard-Anderson JR , Jacob JT , Gottlieb LB. The impact of COVID-19 on multidrug-resistant organisms causing healthcare-associated infections: a narrative review. JAC Antimicrob Resist 2022;5:dlac130. 36601548 10.1093/jacamr/dlac130 PMC9798082

[24] Lastinger LM , Alvarez CR , Kofman A , et al. Continued increases in the incidence of healthcare-associated infection (HAI) during the second year of the coronavirus disease 2019 (COVID-19) pandemic. Infect Control Hosp Epidemiol 2023;44:997–1001. 35591782 10.1017/ice.2022.116 PMC9237489

[25] Weiner-Lastinger LM , Pattabiraman V , Konnor RY , et al. The impact of coronavirus disease 2019 (COVID-19) on healthcare-associated infections in 2020: a summary of data reported to the National Healthcare Safety Network. Infect Control Hosp Epidemiol 2022;43:12–25. 34473013 10.1017/ice.2021.362

[26] National Center for Immunization and Respiratory Diseases (NCIRD), Division of Viral Diseases. Science brief: SARS-CoV-2 and surface (fomite) transmission for indoor community environments. In: *CDC COVID-19 Science Briefs*. Altanta: Centers for Disease Control and Prevention; 2020. 34009771

[27] Interim infection prevention and control recommendations for healthcare personnel during the coronavirus disease 2019 (COVID-19) pandemic. Centers for Disease Control and Prevention website. https://www.cdc.gov/coronavirus/2019-ncov/hcp/infection-control-recommendations.html. Published 2023. Accessed August 4, 2023.

[28] Murthy VH. Confronting health worker burnout and well-being. N Engl J Med 2022;387:577–579. 35830683 10.1056/NEJMp2207252

