# Supplementary material for: Implementation of contact precautions for multidrug-resistant organisms in the post–COVID-19 pandemic era: An updated national Emerging Infections Network (EIN) survey
Source: Infect Control Hosp Epidemiol. 2024 Feb 14;45(6):703–8. doi: 10.1017/ice.2024.11 (PMC11102826; doi:10.1017/ice.2024.11)
Supplement: Howard-Anderson et al. supplementary material 1 — Howard-Anderson et al. supplementary material [file S0899823X24000114sup001.pdf]

Supplemental Materials

Supplemental Table 1: Percentage of respondents whose primary facility uses contact precautions for selected multidrug-resistant organisms by facility type

Organism	Overall (n = 201)	Community hospitals (n=48)	Teaching hospitals[c] (n=127)	VA (n =13)	Other (n=13)
MRSA	133 (66)	30 (63)	82 (65)	13 (100)	8 (62)
VRE	138 (69)	31 (65)	84 (66)	12 (92)	11 (85)
CRE	200 (99)	48 (100)	126 (99)	13 (100)	13 (100)
CRAB [a]	178 (91)	43 (91)	112 (91)	10 (77)	13 (100)
CRPA [b]	163 (85)	42 (89)	98 (82)	10 (77)	13 (100)
ESBL[a]	140 (71)	35 (73)	85 (69)	9 (69)	11 (92)
C. auris	194 (97)	45 (94)	123 (97)	13 (100)	13 (100)

Answers reported as No. (%)

a. Answered by 196 respondents.
b. Answered by 192 respondents.
c. Combined categories of “non-university teaching” and “university” hospitals
