# Supplementary material for: Implementation of contact precautions for multidrug-resistant organisms in the post–COVID-19 pandemic era: An updated national Emerging Infections Network (EIN) survey
Source: Infect Control Hosp Epidemiol. 2024 Feb 14;45(6):703–8. doi: 10.1017/ice.2024.11 (PMC11102826; doi:10.1017/ice.2024.11)
Supplement: Howard-Anderson et al. supplementary material 2 — Howard-Anderson et al. supplementary material [file S0899823X24000114sup002.pdf]

INFECTIOUS DISEASES SOCIETY OF AMERICA EMERGING INFECTIONS NETWORK QUERY:

Use of Contact Precautions for CRE, *Candida auris*, MRSA and VRE

Routine use of gloves and gowns in the care of hospitalized patients (contact precautions, CP) is recommended by CDC to reduce the risk of transmission of multidrug-resistant organisms, including carbapenem-resistant *Enterobacteriaceae* (CRE), *Candida auris*, MRSA and VRE.[1] Use of CP has been questioned and, based on a 2014 EIN, implementation for at least some pathogens in U.S. hospitals has been heterogeneous.[2]

Anecdotal reports have demonstrated a wide variety of practices regarding CP for these organisms, which may have changed in the setting of the COVID-19 pandemic. We are interested in determining practices and beliefs regarding reducing the risk of transmission of multidrug-resistant organisms (MDROs), including CP, for as broad a cross-section of the ID community as possible.

The purpose of this survey is to gauge healthcare facilities' inpatient infection prevention practices for CRE, *C. auris*, MRSA, VRE and other MDROs with particular focus on the use of Contact Precautions.

1. Siegel JD, et al. (2007). 2007 Guideline for Isolation Precautions: Am J Infect Control, 35(10 Suppl 2), S65-164.
2. Russell D, et al. (2016). Routine use of contact precautions for methicillin-resistant *Staphylococcus aureus* and vancomycin-resistant *Enterococcus*: which way is the pendulum swinging. Infect Control Hosp Epidemiol, 37(1):36-40.

.

EMERGING INFECTIONS NETWORK QUERY

Contact Precautions (CP)

Name: ______________________________

__ N/A, not involved in infection prevention. *Stop here*.

1a. Does your primary inpatient facility routinely place patients on CP for the following organisms?

1b. IF CP is employed, please also select all triggers for placement on CP.

	1a. CP?		1b. TRIGGERS		
	Yes	No	Any positive clinical culture	Positive surveillance culture	Pre-existing alert in patient record
CRE	__	__	__	__	__
C. auris	__	__	__	__	__
MRSA	__	__	__	__	__
VRE	__	__	__	__	__

1c. Do any multi-drug resistant Gram-negative organisms other than CRE result in placement on CP?

	Yes	No
Carbapenem-resistant Pseudomonas (CRPA)	__	__
Carbapenem-resistant Acinetobacter (CRAB)	__	__
Extended-spectrum beta-lactamase (ESBL)	__	__
Other: ____________________________	__	__

2. Does your facility perform active surveillance testing for any inpatient population for:

	Yes	No
CRE	__	__
C. auris	__	__
MRSA	__	__
VRE	__	__

3. If you place patients on CP for these pathogens, how long are patients kept on CP?
[Check any that apply]

	N/A	Indefinitely once positive	Until cleared or decolonized	For one year after last positive culture	For specific inpatient encounter only
CRE	__	__	__	__	__
C. auris	__	__	__	__	__
MRSA	__	__	__	__	__
VRE	__	__	__	__	__

Please provide details of duration of isolation, if desired:

Please fax to 319-384-8860 OR 1-866-890-5964

ADJUNCTIVE MEASURES TO REDUCE TRANSMISSION OF RESISTANT ORGANISMS

4. Does your facility perform CHG bathing on any inpatient populations?

__ No, we do not perform CHG bathing on any inpatient population
__ Yes, for all inpatients unless contraindicated
__ Yes, for a subset of patients: *[Select any that apply and specify]*
  __ Pediatric:
  __ Patients with central lines/other implants:
  __ ICU:
  __ Oncology:
  __ Surgical pre-op procedures:
  __ Other:

5. Does your facility use ultraviolet light (UVC) or hydrogen peroxide vapor disinfection in any patient rooms at time of discharge?

__ Yes __ No __ Unsure

6. Does your facility routinely monitor performance of environmental cleaning in any inpatient areas? *[Check any that apply]*

__ Do not monitor
__ Visual inspection
__ Blacklight inspection
__ ATP bioluminescence
__ Unsure

7. For facilities that at one time routinely used CP for MRSA and have since stopped, did you experience change in any of the following? *[Check any that apply]*

__ N/A

	Increased	No change	Decreased	Unsure
a. Hand hygiene compliance	__	__	__	__
b. PPE supply	__	__	__	__
c. Staff satisfaction	__	__	__	__
d. Patient satisfaction	__	__	__	__

8a. Did your institution make changes to CP policies after the COVID pandemic started that are currently still in place?

__ Yes __ No __ Unsure

8b. Do you see your current CP practices changing in the next 12 months or so?

__ Yes __ No __ Unsure

9. Any additional comments about your facility's change in use/current use of Contact Precautions:

Thank you for completing this survey!

Please fax to 319-384-8860 OR 1-866-890-5964
